# Cardiovascular complications and predictors of mortality in hospitalized patients with COVID-19: a cross-sectional study from the Indian subcontinent

**DOI:** 10.1186/s41182-022-00449-w

**Published:** 2022-08-18

**Authors:** Kanhai Lalani, Sneha Seshadri, Jyothi Samanth, Jaimin Jose Thomas, M. Sudhakar Rao, Nisha Kotian, Jijin Satheesh, Krishnananda Nayak

**Affiliations:** 1 Department of Cardiology, Kasturba Medical College, Manipal, Manipal Academy of Higher Education, Manipal, 576104 Karnataka India; 2 Department of General Medicine, Kasturba Medical College, Manipal, Manipal Academy of Higher Education, Manipal, 576104 Karnataka India; 3 Department of Cardiovascular Technology, Manipal College of Health Professions, Manipal Academy of Higher Education, Manipal, 576104 Karnataka India

**Keywords:** COVID-19, Coronavirus, Severe acute respiratory syndrome coronavirus 2 (SARS‑CoV‑2), Cardiovascular complications, Myocarditis, Acute coronary syndrome, Neutrophil–lymphocyte ratio (NLR), Platelet–lymphocyte ratio (PLR)

## Abstract

**Background:**

COVID-19 has spread rapidly across the world, producing significant morbidity and mortality. We investigated the cardiovascular complications and association of laboratory parameters with severity and mortality predictors in COVID-19 hospitalized patients.

**Methods:**

Between May 2020 and June 2021, 730 COVID-19 patients were included in this retrospective observational study in the Coastal Karnataka region of South India. Acute coronary syndrome (ACS), myocarditis, arrhythmias, and all-cause mortality were reported as cardiovascular consequences. Neutrophil/lymphocyte ratio (NLR), platelet/lymphocyte ratio (PLR), serum creatinine, D-dimer, troponin T, N-terminal pro-brain natriuretic peptide (NT-ProBNP), serum ferritin, and serum lactate dehydrogenase (LDH) were among the laboratory parameters measured.

**Results:**

Most common electrocardiogram (ECG) changes were prolonged QTc interval (45.6%) followed by ST-T changes (40.7%) and sinus tachycardia (24.2%). 9.2% patients presented with ACS, with 38.8% having ST-elevation myocardial infarction (STEMI) and 61.2% having non-ST elevation myocardial infarction (NSTEMI). In non-survivors, NLR (*p* < 0.001) and PLR (*p* = 0.001) were significantly higher. Multivariable regression analysis showed that age (OR:1.019, 95% CI 1.003–1.034; *p* = 0.017), acute kidney injury (OR:3.562, 95% CI 1.737–7.301; *p* = 0.001), white blood cell count (WBC) (OR = 1.100, 95% CI 1.035–1.169; *p* = 0.002), platelet count (OR = 0.994, 95% CI 0.990–0.997; *p* = 0.001), PLR (OR = 1.002, 95% CI 1.000–1.004; *p* = 0.023) and severe COVID-19 (OR = 9.012, 95% CI 3.844–21.129; *p* = 0.001) were independent predictors of mortality in COVID-19 patients.

**Conclusions:**

Age, WBC count, neutrophil%, NLR, PLR, creatinine, D-dimer, ferritin, LDH, tachycardia, and lymphocytes% strongly correlated with the severity of the disease. Age, acute kidney injury, elevated WBC count, a greater PLR, low platelet count, and COVID-19 severity were independent predictors of mortality.

**Supplementary Information:**

The online version contains supplementary material available at 10.1186/s41182-022-00449-w.

## Introduction

Severe acute respiratory syndrome coronavirus 2 (SARS‑CoV‑2) has spread very quickly over the globe, causing severe morbidity and mortality. The majority (80%) of COVID-19 patients are asymptomatic or have mild-to-moderate respiratory symptoms, and they usually recover completely with supportive care. COVID-19 is characterized mostly by respiratory symptoms, but some patients may also experience systemic problems including the cardiovascular, neurological, hematological, and immunological dysfunction [1]. Although respiratory failure is a typical COVID-19 clinical symptom [2], the number of COVID-19 related cardiovascular complications is on the rise.

According to the Ministry of health and family welfare of India, the total number of patients with COVID-19 in India has crossed 43 million with 1.21% mortality rate; while Karnataka [3] has total 39 lakhs cases with 1.00% mortality rate. Numerous studies have found that the most frequent risk factors linked to increased severity and death related to COVID-19 were old age, male sex, hypertension, diabetes, smoking, and pre-existing cardiac disease. The case fatality rate is less than 1% in people under the age of 50, while it is close to 15% in people over the age of 80 [4, 5]. According to Young et al. [6], Fan et al. [7], Yang et al. [8] studies, greater severity of COVID-19 and mortality are often linked to elevated levels of C-reactive protein (CRP), serum ferritin, lactate dehydrogenase (LDH), elevated D-dimer, and raised prothrombin time (PT).

COVID-19 has the potential to worsen the prognosis of patients with established CVD due to reduced cardiorespiratory reserve, worsening of pre-existing CVD due to systemic inflammatory response, or triggering new onset cardiac disease [9]. Mortality remains 2.3% in absence of comorbidities, compared to 6% for hypertensives, 7.3% for diabetics, and 10.5% for people with pre-existing CVD [10, 11]. The most frequently (8–30%) reported abnormality in COVID-19 is acute myocardial injury accompanied by considerable elevation of cardiac troponins [12]. Overall arrhythmias were reported in around 16.7% patients; 44.4% in severe cases and 8.9% in mild cases [1, 2, 13]. An Indian study by Kunal et al. [14] reported ECG changes such as prolonged QTc (17.6%), sinus tachycardia (16.9%), first degree AV block (4.6%), ventricular arrhythmias (1.8%) and sinus bradycardia (0.9%). Acute cardiac injury (25.9%), heart failure (3.7%), cardiogenic shock (3.7%), acute coronary syndrome (3.7%), and myocarditis (2.8%) were observed cardiovascular complications. Cardiovascular problems during hospitalization have a detrimental effect on the patient's in-hospital outcome. One of the primary reasons for mortality in COVID-19 patients is viral myocarditis and cardiac injury. In Chinese studies [1, 2, 10], the overall case fatality rate was 2–5%, while the death rate in patients with cardiovascular complications was 10.5%.

Kerala, a state in southern India, announced the first case of COVID-19 in the country on January 30, 2020 [15]. Serum ferritin, C-reactive protein, lactic acid dehydrogenase, and neutrophil-to-lymphocyte ratio values were significantly higher in a study from Kerala [15] for patients who were critically ill, whereas age and multimorbidity were among the major risk factors for death in hospitalized patients with COVID-19. Cardiovascular complications of COVID-19 and its predictors have not been described much in the Indian population. The lack of data moreover in the south Indian population encouraged us to conduct additional research to investigate the differences in the incidence of cardiovascular complications and outcomes among COVID-19 patients compared to other parts of India and globally. This was a retrospective cross-sectional study to demonstrate the cardiovascular complications, hemocytometric and biochemical parameters, and association of laboratory parameters with severity and outcomes in patients with COVID-19 patients admitted to a tertiary health hospital in coastal Karnataka region of southern India. To the best of our knowledge, this is the largest study from Indian population looking at cardiovascular outcomes, all-cause mortality, and predictors in hospitalized patients with COVID-19.

## Methods

### Study design and study area

**Fig. 1:**
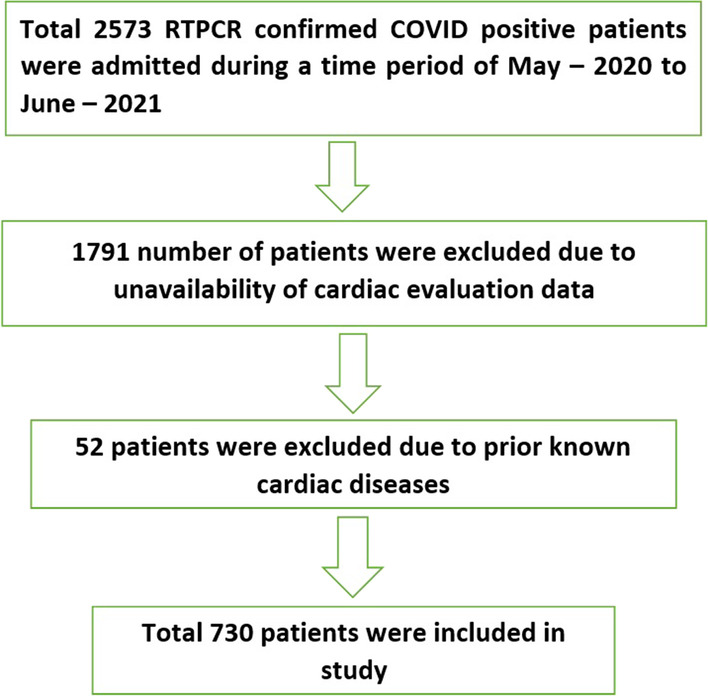
Flowchart of patient inclusion and exclusion in the study

This retrospective cross-sectional study was conducted at a tertiary care hospital in the Coastal Karnataka region of South India between May-2020 and June-2021 among patients with COVID-19. Eligible patients (730) were included in the study (Fig. 1).

### Study population

This study included reverse transcription-polymerase chain reaction (RT-PCR)-confirmed COVID-19 hospitalized patients older than 18 years who underwent cardiac assessment through electrocardiogram and echocardiography. From May-2020 to June-2021, this study included only patients who were exposed to COVID-19 for the first time and had no prior history of COVID-19 infection. During this time, all RT-PCR confirmed positive COVID-19 patients, regardless of severity, were isolated and admitted to the hospital because home isolation was not permitted as per local state policy. All patients who were admitted to the hospital for COVID-19-related illnesses had electrocardiograms. Patients with COVID-19 had cardiac evaluation through echocardiography at the discretion of their treating physician.

Patients with a prior history of myocardial infarction with LV dysfunction, pre-existing structural heart disease, and those without an echocardiogram were excluded.

### Data collection

Baseline characteristics of patients such as age, gender, blood pressure on admission, comorbidities, and COVID-19 severity were noted. All patient data were obtained from their case sheet records and blood investigation reports. The number of patients who were treated with COVID-19-related specific medications such as steroids and remdesivir were also noted. However, as per our local hospital policy, hydroxychloroquine, tocilizumab, and other specific drugs were not approved and not used during this study period. On admission, laboratory investigations such as hemoglobin (g/dL), white blood cell (WBC) count (× 10^3^/µL), neutrophil count (%), lymphocyte count (%), neutrophil/lymphocyte ratio, platelet count (× 10^3^/µL), platelet/lymphocyte ratio, serum creatinine (mg/dL), lipid profile, D-dimer (g/L), serum troponin T (ng/mL), N-terminal pro-brain natriuretic peptide (NT-ProBNP) (ng/L), serum ferritin (ng/mL), and serum lactate dehydrogenase (LDH) (U/L) were noted from case sheet records.

Baseline ECG parameters such as heart rate, ST changes, conduction blocks, PR interval, QTc interval, and arrhythmias were recorded.

Baseline echocardiography parameters such as end-diastolic dimension, end-systolic dimension, left ventricular (LV) ejection fraction, fractional shortening, regional wall motion abnormalities, valvular regurgitation, right ventricular systolic pressure (RVSP), tricuspid annular plane systolic excursion (TAPSE), right ventricular (RV) dysfunction, and pericardial effusion were noted.

Cardiovascular complications such as acute coronary syndrome (ACS), myocarditis, arrhythmias, cardiogenic shock, pericardial effusion, intracardiac thrombus, and all-cause in-hospital mortality were noted.

### Study variables

#### Case definition

- COVID-19 cases were classified as follows: (AIIMS/ICMR guideline, Ministry of health and family welfare, government of India) [16].
  - Mild: Upper respiratory tract symptoms and/or fever without shortness of breath or hypoxia.
  - Moderate: Any one of:
    1. Respiratory rate ≥ 24/min, breathlessness.
    2. SpO_2_: 90–93% on room air.
  - Severe: any one of:
    1. Respiratory rate ≥ 30/min, breathlessness.
    2. SpO_2_: < 90% on room air.

#### Electrocardiogram (ECG) definitions

- Sinus bradycardia [17] was defined as heart rate < 60/min and sinus tachycardia was defined as heart rate > 100/min.
- The ST-segment deviation was measured as the height difference (in mm) between the J-point and the isoelectric line (TP segment).
- Corrected QT interval was measured with Bazett’s formula [18]. QTc = QT interval/√RR. RR is the interval from the onset of one QRS complex to the onset of the next QRS complex. QTc interval > 460 ms in females and > 450 ms in males was defined as a prolonged QTc interval [19].
- Atrial fibrillation [20] was diagnosed by a 12-lead ECG showing heart rhythm with no discernible repeating P waves and irregular RR intervals (when atrioventricular conduction is not impaired).

#### Echocardiogram definitions

- Pulmonary arterial hypertension (PAH) was graded on basis of the right ventricular systolic pressure (RVSP). Significant PAH was considered when RVSP > 50 mmHg.
- RV dysfunction was diagnosed when TAPSE < 16 mm [21].
- Pericardial effusion was diagnosed as an echo-free space between the two layers of the pericardium and > 10 mm effusion was considered significant.

#### Complications

- ACS includes ST-elevation myocardial infarction (STEMI) and non-ST elevation myocardial infarction (NSTEMI) in our study.
  I. STEMI [21] was defined as “New ST-elevation at the J-point in 2 contiguous leads: ≥ 1 mm in all leads except V2–V3; in leads V2–V3 ≥ 2 mm in men ≥ 40 years, ≥ 2.5 mm in men < 40 years, or ≥ 1.5 mm in women regardless of age.
  II. NSTEMI [21] was defined as “New horizontal or downsloping ST-depression ≥ 0.5 mm in 2 contiguous leads and/or T inversion > 1 mm in 2 contiguous leads with prominent R wave.”
- Myocarditis [22] was defined as:
  1. New onset of LV/RV dysfunction in patients with a previously normal echocardiographic study on admission.
  2. LV dysfunction with evidence of myocardial necrosis (Trop > 0.1).
- Cardiogenic shock [23] was defined as “persistent hypotension (SBP < 90 mmHg) even with inotropic support leading to decrease in cardiac output and tissue hypoperfusion in the presence of adequate intravascular volume.”
- Pulmonary embolism was diagnosed by CT pulmonary angiography in suspected patients.
- All-cause mortality or non-survivors was expressed as the total number of deaths due to COVID-19 illness during this study period. It had included death due to any causes either cardiac or non-cardiac.

### Protocol of management

Many patients with COVID-19 were treated with remdesivir and steroids apart from the standard supportive treatment during this study period as per the treating physician’s discretion. Injection remdesivir was given 200 mg intravenous (IV) on day 1 followed by 100 mg IV once a day for the next 4 days. Injection methylprednisolone was given 0.5–2 mg/kg IV in 2 divided doses for a duration of 5–10 days [16].

### Statistical analysis

Data analysis was performed using SPSS software version 25. Categorical variables are shown as frequency and percentages. Continuous variables such as age, blood pressure, hemoglobin, heart rate, QTc interval are presented as mean (±SD) and WBC count, neutrophil, lymphocyte, NLR, platelet count, PLR, serum creatinine, D-dimer, troponin, NT-ProBNP, ferritin, LDH are presented as median (interquartile range [IQR]). The Chi-square test was used to find the association between two categorical variables. ANOVA test was performed to compare variances across the means (or average) of more than two groups. Multivariate analysis was performed to find independent predictors of all-cause mortality with biochemical and hematological parameters. Hemocytometric parameters were also compared among survivors and non-survivors. A *p*-value of less than 0.05 was considered statistically significant.

## Results

A total of 2573 patients with COVID-19 were admitted to our hospital from May 15th, 2020 to June 5th, 2021. Of these, only 730 patients were included in our study (Fig. 1).

### Baseline characteristics

Baseline characteristics such as age, sex, blood pressure, comorbidities, and COVID-19 severity are presented in Table 1. There were 508 males (69.5%) and 222 females (30.3%) among the 730 patients. 407 (55.8%) had diabetes mellitus, 349 (47.8%) had hypertension, 92 (12.6%) had chronic kidney disease (CKD,) and 35 (4.8%) had a history of cerebrovascular accident (CVA). 309 (42.3%) patients had mild COVID-19, 139 (19%) patients had moderate COVID-19, and 282 (38.6%) patients had severe COVID-19. 248 (34%) patients were treated with steroids, 199 (27.3%) were treated with remdesivir, and 147 (20.1%) were treated with both steroids and remdesivir.

**Table 1:** Baseline characteristics

Parameters	Value
Mean age (years, mean ± SD)	58.81 ± 16.83
Gender	
Male (*n*, %)	508 (69.5%)
Female (*n*, %)	222 (30.3%)
Blood pressure	
SBP (mmHg, mean ± SD)	125.25 ± 22.00
DBP (mmHg, mean ± SD)	77.09 ± 12.28
Risk factors (*n*, %)	
DM	407 (55.8%)
HTN	349 (47.8%)
CKD	92 (12.6%)
CVA	35 (4.8%)
COVID-19 severity (*n*, %)	
Mild	309 (42.3%)
Moderate	139 (19.0%)
Severe	282 (38.6%)
Specific treatment (*n*, %)	
Remdesivir	199 (27.3%)
Steroid	248 (34.0%)
Remdesivir and steroid both	147 (20.1%)

*CKD* chronic kidney disease, *CVA* cerebrovascular accident, *DBP* diastolic blood pressure, *DM* diabetes mellitus, *HTN* hypertension, *SBP* systolic blood pressure

### Laboratory investigations

Laboratory investigations are presented in Table 2. Troponin T and NT-proBNP levels were only available in 490 (67.1%) and 452 (61.9%) patients, respectively. 554 (75.9%) patients had D-dimer, 609 (83.4%) patients had ferritin, and 607 (83.2%) patients had LDH data available.

**Table 2:** Laboratory investigations

Parameters	Value
Complete blood count (CBC)	
Hemoglobin (g/dL, mean ± SD)	12.0 ± 4.0
WBC count (× 10^3^/µL, median, Q1–Q3)	9.00 (6.20–12.50)
Neutrophil (%, median, Q1–Q3)	77.60 (67.07–88.22)
Lymphocyte (%, median, Q1–Q3)	11.90 (6.50–19.20)
Neutrophil/lymphocyte ratio (median, Q1–Q3)	6.50 (3.48–12.68)
Platelet count (× 10^3^/µL, median, Q1–Q3)	226.50 (163.25–310.75)
Platelet/lymphocyte ratio (median, Q1–Q3)	220.00 (135.00–365.00)
Serum creatinine (mg/dL, median, Q1–Q3)	0.90 (0.78–1.39)
D-dimer (g/L, median, Q1–Q3)	0.90 (0.40–2.70)
Cardiac Troponin T (ng/mL, median, Q1–Q3)	0.028 (0.009–0.925)
NT-ProBNP (ng/L, median, Q1–Q3)	725 (214–2890)
Serum ferritin (ng/mL, median, Q1–Q3)	506 (208–1095)
Serum LDH (U/L, median, Q1–Q3)	418 (292–605)

*LDH* lactate dehydrogenase, *NT-ProBNP* N-terminal pro-brain natriuretic peptide, *WBC* white blood cell

### ECG abnormalities

ECG abnormalities are presented in Table 3. The most common ECG abnormalities were prolonged QTc interval seen in 283 (45.6%) patients followed by ST-T changes in 297 (40.7%) and sinus tachycardia in 177 (24.2%) patients. There were 172 (27.7%) males and 111 (17.9%) females among the 283 individuals with prolonged QTc. Atrial fibrillation was observed only in 18 (2.5%) patients. Only two individuals had third-degree AV block, both of whom had associated inferior wall myocardial infarction (IWMI).

**Table 3:** ECG abnormalities

Parameters	*n* (%)
QTc prolongation (*n*, %)	283 (45.6%)
Male (*n*, %)	172 (27.7%)
Female (*n*, %)	111 (17.9%)
ST-T changes (*n*, %)	297 (40.7%)
Sinus tachycardia (*n*, %)	177 (24.2%)
Sinus bradycardia (*n*, %)	34 (4.7%)
Atrial fibrillation (*n*, %)	18 (2.5%)
VPCs (*n*, %)	33 (4.5%)
Heart block (*n*, %)	
First degree	8 (1.1%)
Second degree	4 (0.5%)
Third degree	2 (0.3%)
Bundle branch block (*n*, %)	
Absent	614 (84.1%)
LBBB	11 (1.5%)
RBBB	21 (2.9%)

*LBBB* left bundle branch block, *RBBB* right bundle branch block, *VPCs* ventricular premature complexes

### Cardiovascular complications

Cardiovascular complications and comorbidities associated with COVID-19 severity are presented in Table 4. There were 67 (9.2%) patients who presented with ACS, with 26 and 41 patients having STEMI and NSTEMI, respectively. Anterior wall myocardial infarction (AWMI) was the most common [14 (20.9%)], followed by inferior wall myocardial infarction (IWMI) [10 (14.93%)], and infero-posterior wall myocardial infarction (IPWMI) [2 (2.9%)]. 58.2% of STEMI patients presented with the occluded vessel (TIMI 0). Hypertension emerged as a risk factor (*p* = 0.05) for acute coronary syndrome among COVID-19 individuals. Other risk factors including DM and history of CVA were not significant (Additional file 1: Table S1).

**Table 4:** Cardiovascular complications and comorbidities among the severity of COVID-19 patients

Complications	Total (*n* = 730)	Mild (*n* = 309)	Moderate (*n* = 139)	Severe (*n* = 282)	*p*-value
All-cause mortality (*n*, %)	166 (22.7%)	19 (6.1%)	4 (2.9%)	143 (50.7%)	< 0.001
ACS (*n*, %)	67 (9.2%)	32 (10.4%)	11 (7.9%)	24 (8.5%)	0.959
AWMI	14 (20.9%)	6 (18.8%)	4 (36.4%)	4 (16.7%)	
IWMI	10 (14.9%)	6 (18.8%)	1 (9.1%)	3 (12.5%)	
IPWMI	2 (2.9%)	2 (6.3%)	0 (0%)	0 (0%)	
NSTEMI	41 (61.2%)	18 (56.3%)	6 (54.6%)	17 (70.8%)	
Myocarditis (*n*, %)	60 (8.2%)	25 (8.1%)	12 (8.6%)	23 (8.2%)	0.968
AF (*n*, %)	18 (2.5%)	11 (3.6%)	1 (0.7%)	6 (2.1%)	0.03
Cardiogenic shock (*n*, %)	3 (0.4%)	1 (0.3%)	1 (0.7%)	1 (0.4%)	0.603
Acute kidney injury (*n*, %)	70 (9.6%)	16 (5.2%)	8 (5.8%)	46 (16.3%)	< 0.001
PE (*n*, %)	25 (3.4%)	10 (3.2%)	4 (2.9%)	11 (3.9%)	0.896
Intracardiac thrombus (*n*, %)	4 (0.5%)	1 (0.3%)	1 (0.7%)	2 (0.7%)	0.681
DM (*n*, %)	407 (55.8%)	139 (45%)	84 (60.4%)	184 (65.2%)	< 0.001
HTN (*n*, %)	349 (47.8%)	129 (41.7%)	71 (51.1%)	149 (52.8%)	0.018
CKD (*n*, %)	92 (12.6%)	34 (11%)	12 (8.6%)	46 (16.3%)	0.05
CVA (*n*, %)	35 (4.8%)	16 (5.2%)	5 (3.6%)	14 (5%)	0.831

*ACS* acute coronary syndrome, *AF* atrial fibrillation, *AWMI* anterior wall myocardial infarction, *IWMI* inferior wall myocardial infarction, *IPWMI* infero-posterior wall myocardial infarction, *NSTEMI* non-ST segment elevation myocardial infarction, *PE* pulmonary embolism

Myocarditis was found in 60 (8.2%) patients; however, the numbers were comparable across severity groups. Significant PAH was seen in 13 (1.8%) patients, whereas significant pericardial effusion was found in only 2 (0.3%) patients. Symptomatic pulmonary embolism was seen in 25 (3.4%) patients, while intracardiac thrombus was observed in only 4 (0.54%) patients, with no significant difference based on COVID-19 severity. In the severe COVID-19 group compared to the mild or moderate group, there were substantially more individuals with DM (*p* < 0.001), HTN (*p* = 0.018), and CKD (*p* = 0.05).

Out of 730 patients, 166 (22.7%) patients died due to COVID-19-related complications. The severe COVID-19 group had significantly higher mortality compared to the mild disease group [143 (50.7%) versus 19 (6.1%) in severe and mild COVID-19 group, respectively]. Diabetes, hypertension, and CVA were comparable among survivors and non-survivors, whereas CKD patients had significantly higher all-cause mortality (*p* = 0.003) (Additional file 1: Table S2).

Baseline demographic variables, laboratory investigations, and ECG parameters were compared among mild, moderate, and severe COVID-19 patients. There is a significant difference in age, WBC count, neutrophil %, neutrophil–lymphocyte ratio (NLR), platelet lymphocyte ratio (PLR), serum creatinine, D-dimer, serum ferritin, serum LDH and heart rate among the groups where higher values were observed in severe COVID-19, and lower values among mild COVID-19. Significantly higher lymphocyte % values were observed in the mild COVID-19 compared to the severe COVID-19 [16.6 (8.97–24.67) in the mild group versus 8.8 (5–14.2) in the severe group, *p* < 0.001] (Table 5).

**Table 5:** Demographic data, laboratory investigations and ECG changes among the severity of COVID-19 patients

Parameters	Severity of COVID-19 [mean ± SD] or [median (Q1–Q3)]			*p* value
	Mild (*n* = 309)	Moderate (*n* = 139)	Severe (*n* = 282)	
SBP (mmHg)	125.78 ± 23.19	125.26 ± 21.16	124.68 ± 21.19	0.851
DBP (mmHg)	78.44 ± 11.94	76.14 ± 10.62	76.17 ± 13.25	0.07
Age (years)	57.40 ± 14.93	61.69 ± 23.36	58.90 ± 14.68	0.04
Hb (g/dL)	12.43 ± 6.73	12.30 ± 2.05	12.18 ± 2.52	0.808
WBC count (× 10^3^/µL)	8.10 (5.90–11.00)	8.20 (5.90–11.12)	10.80 (7.17–14.20)	< 0.001
Neutrophil%	71.55 (61.10–81.02)	77.00 (68.62–84.65)	82.05 (74.62–88.80)	< 0.001
Lymphocyte%	16.60 (8.97–24.67)	12.20 (7.42–18.52)	8.80 (5.00–14.2)	< 0.001
NL ratio	4.32 (2.45–8.92)	6.28 (3.81–11.54)	9.25 (5.29–17.41)	< 0.001
Platelet count (× 10^3^/µL)	234.00 (170.50–310.50)	221.50 (159.50–345.00)	224.00 (161.00–300.50)	0.791
PL ratio	177.65 (121.55–303.97)	223.03 (148.4–315.55)	283.14 (155.05–453.19)	< 0.001
Creatinine (mg/dL)	0.93 (0.78–1.29)	1.00 (0.3–1.92)	1.06 (0.77–1.62)	0.045
D-dimer (g/L)	0.6 (0.3–2.15)	0.6 (0.3–1.92)	1.4 (0.6–4.2)	< 0.001
Cardiac Troponin T (ng/mL)	0.034 (0.009–0.109)	0.016 (0.070–0.070)	0.028 (0.012–0.088)	0.104
NT-ProBNP (ng/L)	715.50 (149.75–2644.75)	609.00 (215.50–3809.00)	764.50 (245.75–2829.50)	0.583
Ferritin (ng/mL)	285.15 (146.45–690.85)	487 (240.50–869.00)	726.35 (336.75–1444.00)	< 0.001
LDH (U/L)	299.00 (230.50–435.50)	398.00 (314.00–531.00)	534.00 (336.75–753.00)	< 0.001
HR (/min)	87.09 ± 20.54	90.22 ± 17.68	95.28 ± 31.72	< 0.001
QTc (ms)	437.21 ± 94.94	453.72 ± 74.33	441.87 ± 96.84	0.244

*DBP* diastolic blood pressure, *Hb* hemoglobin, *HR* heart rate, *LDH* lactate dehydrogenase, *NLR* neutrophil–lymphocyte ratio, *NT-ProBNP* N-terminal pro-brain natriuretic peptide, *PLR* platelet–lymphocyte ratio, *SBP* systolic blood pressure, *WBC* white blood cell

A significantly higher mean heart rate was observed in the severe COVID-19 compared to the mild COVID-19 group [(95.28 ± 31.72) in the severe group versus (87.09 ± 20.54) in the mild group, *p* < 0.001] (Table 5).

In non-survivors, NLR was significantly higher than in survivors [10.08 (5.35–18.78) in non-survivors versus 5.7 (3.2–11.12) in survivors, *p* < 0.001]. Similarly, PLR in non-survivors was significantly higher than in survivor group [289.13 (143.57–485.59) in non-survivors versus 207.48 (134.12–339.24) in survivors, *p* = 0.001] (Additional file 1: Table S3).

Multivariable regression analysis showed age (OR = 1.019), acute kidney injury (OR = 3.562), WBC count (OR = 1.100), platelet count (OR = 0.994), PLR (OR = 1.002), and severe COVID-19 (OR = 9.012) were independent predictors of mortality in COVID-19 patients (Table 6).

**Table 6:** Multivariable regression analysis in the prediction of all-cause mortality

Parameters	Adjusted odds ratio Exp (B)	95.0% CI for Exp (B)		
		Lower	Upper	*p* value
Age	1.019	1.003	1.034	0.017
DM	1.062	0.607	1.859	0.834
HTN	0.798	0.453	1.406	0.435
CKD	1.734	0.797	3.775	0.165
AF	1.461	0.392	5.449	0.572
EF	0.997	0.966	1.030	0.874
Myocarditis	1.250	0.400	3.899	0.701
Acute kidney disease	3.562	1.737	7.301	0.001
WBC count	1.100	1.035	1.169	0.002
Neutrophil %	0.990	0.953	1.029	0.617
Neutrophil–lymphocyte ratio	1.003	0.961	1.047	0.892
Lymphocyte %	0.944	0.883	1.010	0.093
Platelet count	0.994	0.990	0.997	0.001
Platelet–lymphocyte ratio	1.002	1.000	1.004	0.023
Ferritin	1.000	1.000	1.001	0.090
LDH	1.001	1.000	1.001	0.232
Cardiac Troponin T	1.017	0.963	1.074	0.541
D-dimer	1.060	0.969	1.159	0.206
Severe COVID-19	9.012	3.844	21.129	0.001

*AF* atrial fibrillation, *CI* confidence interval, *CKD* chronic kidney disease, *DM* diabetes mellitus, *EF* ejection fraction, *HTN* hypertension, *LDH* lactate dehydrogenase, *WBC* white blood cell

## Discussion

Severe acute respiratory syndrome coronavirus 2 (SARS‑CoV‑2) was identified in 2019 and resulted in a pandemic acute respiratory illness, called COVID-19. Clinical symptoms can be varied from fever, cough, fatigue, headache, diarrhea, hemoptysis, dyspnea, ageusia, and anosmia [24]. Cardiovascular manifestations play a significant role in predicting mortality among COVID-19 patients. Our study aims to investigate various cardiac manifestations in COVID-19 patients and to determine the predictors of outcomes in patients with COVID-19.

In our study, the average age was 58.8 years, with a male predominance (69.5%), which was comparable with other studies [8, 25–28]. Mean age was significantly higher in the severe group, but clinically not relevant. Moreover, many COVID-19 patients had underlying comorbidities such as diabetes (55.8%), hypertension (47.8%), CVA (4.8%), and CKD (12.6%). Patients with hypertension, diabetes, cerebrovascular illness, and prior cardiovascular disease are more likely to suffer cardiovascular complications such as acute cardiac damage and mortality as a result of COVID-19 infection [8, 25, 26, 29, 30]. Similarly, patients with diabetes (*p* < 0.001), hypertension (*p* = 0.018) and CKD (*p* = 0.05) were significantly more prevalent in severe COVID-19 group in our study.

The most common ECG findings in our study were QTc prolongation (45.6%), ST-T changes (40.7%), and sinus tachycardia (24.2%). Prolonged QTc interval was found in 45.6% of our study group, although no malignant arrhythmia was found; that was similar to the findings of Luca et al. [31]. In our study, we found a higher proportion of individuals with prolonged QTc, which might be linked to the frequent prophylactic use of hydroxychloroquine or antibiotics like azithromycin [32]. Male patients had a higher proportion of prolonged QTc intervals (27.7%) than female patients (17.9%), which may be related to the higher proportion (69.5%) of male patients in the study group.

In the study by Yina Wang et al. [33], ST-T changes were seen in 32.6%, sinus tachycardia in 12.5%, and atrial fibrillation in 6.6% of patients. In patients with COVID-19, Luca et al. [31] discovered a link between aberrant ECG and serious adverse events. New-onset atrial fibrillation was strongly linked to an increased risk of significant adverse outcomes; however, there was no link between prolonged QTc interval and ventricular arrhythmias. Similarly, we did not find any correlation between atrial fibrillation and outcomes. Changes in ST-T levels may be linked to dyselectrolytemia, coronary artery disease, hypertension, or myocardial damage caused by the COVID-19 virus. Due to the lack of a baseline ECG before the onset of COVID-19, aberrant ST-T changes cannot be regarded as typical COVID-19 findings, but they are nevertheless useful in diagnosing and determining the severity of the myocardial injury. Sinus tachycardia can be caused by fever, hypoxia, systemic inflammatory response, sympathetic activation, myocardial injury, or heart failure. In our study, unadjusted heart rate was significantly higher in the severe COVID-19 group compared to mild and moderate COVID-19 patients. Zhe et al. [34] showed that COVID-19 patients with myocardial injury frequently exhibit sinus tachycardia. Atrial fibrillation was identified during the hospital stay in just 2.5% of our patients, which was most likely owing to the underdiagnosis of atrial fibrillation because no patients had a Holter investigation. ECG was used to diagnose only clinically symptomatic AF episodes.

ACS is a well-known COVID-19 consequence, and its pathophysiology might be linked to the virus's hypercoagulable condition, which causes coronary artery thrombosis. In our study, 67 (9.2%) individuals presented with ACS, with 26 (39%) patients having STEMI and 41 (61.19%) having NSTEMI. Among STEMI, AWMI was the most prevalent followed by IWMI. 58.2% of STEMI patients presented with the occluded vessel (TIMI 0). Diabetes, CKD, and CVA were comparable in patients with and without acute coronary syndrome, whereas hypertension (*p* = 0.05) emerged as a prevalent risk factor in patients with COVID-19 and ACS. Giulio G. Stefanini et al. [35] investigated 28 COVID-19 patients with STEMI. 85.7% of patients had STEMI at the time of admission, and 14.3% had STEMI while in the hospital. 78.6% reported classic chest discomfort. 60.7% of those who needed revascularization had a culprit lesion, whereas 39.3% did not have obstructive coronary artery disease. Bangalore et al. [36] reported 18 COVID-19 patients who presented with STEMI. 83% of the patients were males. Only 33% of people complained of chest discomfort. 67% of the participants had obstructive CAD, and 56% underwent percutaneous intervention. 13 patients (72%) had in-hospital mortality.

Infection with COVID-19 can induce cardiac damage in up to 30% of patients and is related to greater disease severity [2]. COVID-19 can induce myocarditis by a combination of direct cellular damage and cytotoxic responses mediated by T cells [37]. Fever, shortness of breath, coughing, and chest pain are the most prevalent presenting symptoms. Myocarditis causes increased cardiac biomarkers such as cardiac troponins, non-specific ST-segment and T-wave alterations, and atrioventricular blocks on ECG, which have been linked to more severe disease and were predictive of mortality in COVID-19. This cardiac damage is associated with increased interleukin-6 and C-reactive protein and may be caused by systemic inflammation and cytokine storm caused by viral infection [37].

Myocarditis was found in 60 (8.2%) of our patients, and there was no significant difference across COVID-19 severity groups. The real incidence of myocarditis may be underestimated because a considerable number of individuals (1791 patients) were excluded during initial screening due to a lack of echocardiogram data. However, most of the patients who were excluded from the study due to the unavailability of echocardiogram data were mild cases and asymptomatic. So, there are very less chances of missing clinical myocarditis in those cases.

Jamie et al. [37] reported a meta-analysis of 31 papers that comprised 51 patients; 12 cases had confirmed myocarditis, while 39 had possible myocarditis. Daniels et al. [38] found a 2.3% overall prevalence of clinical or subclinical myocarditis (0–7.6%). Deng et al. [39] showed a 12.5% incidence of myocarditis. Histopathological examination is the gold standard in the diagnosis of myocarditis. Endomyocardial biopsy is an invasive technique, it is seldom used in clinical settings, especially in COVID-19-positive patients who are at risk of infection [37]. Cardiac MRI (CMR) can also be used to establish a clinical diagnosis. Due to the difficulty of acquiring the scan during an infectious illness epidemic, the majority of patients had no CMR despite having suggestive findings of myocarditis on serology, ECG, and echocardiography [37].

Neutrophil–lymphocyte ratio (NLR) and platelet–lymphocyte ratio (PLR) are two biomarkers that give essential information about the state of the inflammatory process and may be obtained readily from standard laboratory investigations. White blood cell (WBC) counts, and neutrophil percent increased significantly with the severity of COVID-19 in our study. NLR was significantly higher in severe disease compared to mild disease. The percentage of lymphocytes dropped as the severity of COVID-19 increased, whereas the platelet–lymphocyte ratio was significantly higher in severe COVID-19 compared to milder disease. The NLR and PLR were found to be independent risk factors for the severity and mortality among COVID–19 patients in recent studies [40–43].

The NLR and PLR ratios were significantly higher in non-survivors compared to survivors in our study. Increased neutrophils reflect the severity of the inflammatory response, whereas reduced lymphocytes reflect the degree of the virus's immunological imbalance and immune-escape strategy [44, 45]. The decline in lymphocytes might be attributed to virus infection or antiviral responses that suppress myelocytes, weakening the immune system even further. The cytokine storm is caused by unchecked and overreactive immune responses, resulting in widespread alveolar damage or multi-organ failure, and eventually death from COVID-19 [8, 40].

Lab indicators such as elevated creatinine, D-dimer, ferritin, and LDH were shown to be related to the severity of COVID-19 in our study. Serum creatinine levels in the severe disease group were substantially greater than those in the milder group. A multivariable regression analysis revealed that COVID-19 patients with acute renal damage had a greater chance of mortality [12, 13, 46]. Serum D-dimer levels were significantly higher in the severe COVID-19 group compared to the mild disease group similar to previously published studies [12, 47, 48]. Serum ferritin levels were significantly higher in the severe COVID-19 group compared to the mild disease group which was in accordance with previously published studies [12, 48, 49]. Multivariable regression analysis, however, revealed that ferritin was not an independent predictor of mortality. Though the specific cause of hyperferritinemia is unknown, higher proinflammatory cytokines such as TNF-alpha, IL-1, IL-6, etc., in COVID-19 patients are thought to enhance serum ferritin production [49]. Serum LDH levels were significantly higher in the severe COVID-19 group compared to the mild disease group which is comparable with other studies [12, 13, 48, 50]. However, in our study LDH levels did not correlate with all-cause mortality. Troponin T and NT-proBNP levels were available in 490 (67.1%) and 452 (61.9%) patients, respectively, in our study. Their levels were comparable across all groups and were not predictive of mortality in our study. Many studies published previously [13, 50–52] showed higher troponin values were associated with poor prognosis and mortality in COVID-19 patients.

### Limitations

A few limitations in our retrospective study deserve a mention. Due to the lack of cardiac echocardiogram data, a considerable number of patients (1791) were eliminated from the retrospective analysis. Second, the number of atrial fibrillation patients was low and so the association between COVID-19 and atrial fibrillation could not be determined. Third, cardiac biomarkers like troponin and NT-proBNP were only accessible in around 70% of patients and so the results could not be properly interpreted. Fourth, actual cases of myocarditis may be underestimated due to diagnostic dilemmas in certain cases and the lack of echocardiograms and cardiac biomarkers. Fifth, hemocytometric parameter values were recorded on admission but not serially followed up to assess serial rise or fall. This may lead to the misunderstanding of the predictions of biomarkers in all-cause mortality in COVID-19. A long-term prospective study with a large number of patients is required to overcome these limitations.

## Conclusion

In our study, QTc prolongation was the most common ECG abnormality followed by ST-T changes and sinus tachycardia. Myocarditis was detected in a substantial number of patients. Among STEMI, AWMI was the most common, followed by IWMI. Acute kidney injury, high WBC count, greater platelet–lymphocyte ratio, and severe COVID-19 were all independent predictors of mortality. Elevated WBC count, neutrophil%, NL ratio, PL ratio, creatinine, D-dimer, ferritin, LDH levels, and reduced lymphocytes% strongly correlated with the severity of the disease. Our study highlights the importance of laboratory abnormalities along with electrocardiographic findings and clinical associates in the identification of prognostically poor COVID-19 patient.

## Supplementary Information

**Additional file 1. Supplementary Table 1**. Risk factors associated with acute coronary syndrome.** Supplementary Table 2**. Risk factors associated with all-cause mortality.** Supplementary Table 3**. The value of NLR and PLR for survivors and non-survivors of COVID-19.

## Data Availability

The datasets used and/or analyzed during the current study are available from the corresponding author upon reasonable request.
