# Supplementary material for: Cardiovascular complications and predictors of mortality in hospitalized patients with COVID-19: a cross-sectional study from the Indian subcontinent
Source: Trop Med Health. 2022 Aug 18;50:55. doi: 10.1186/s41182-022-00449-w (PMC9385411; doi:10.1186/s41182-022-00449-w)
Supplement: Supplementary file 1 — Additional file 1. Supplementary Table 1. Risk factors associated with acute coronary syndrome. Supplementary Table 2. Risk factors associated with all-cause mortality. Supplementary Table 3. The value of NLR and PLR for survivors and non-survivors of COVID-19. [file 41182_2022_449_MOESM1_ESM.docx]

**Supplementary table 1: Risk factors associated with acute coronary syndrome.**

| **Risk factors** | **Acute coronary syndrome**  **(n,%)** | | **p-value** |
| --- | --- | --- | --- |
|  | **YES**  **(n=67)** | **NO**  **(n=662)** |  |
| DM (n,%) | 39 (58.2%) | 368 (55.5%) | 0.70 |
| HTN (n,%) | 40 (59.7%) | 309 (46.6%) | 0.05 |
| CKD (n,%) | 8 (11.9%) | 84 (12.7%) | 0.525 |
| CVA (n,%) | 3 (4.5%) | 32 (4.8%) | 1.00 |

Abbreviations: **CKD:** Chronic kidney disease, **CVA:** Cerebrovascular accident, **DM:** Diabetes mellitus, **HTN:** Hypertension.

**Supplementary table 2: Risk factors associated with all-cause mortality**

| **Risk factors** | **All-cause mortality (n,%)** | | **p-value** |
| --- | --- | --- | --- |
|  | **YES**  **(Non-survivors)**  **(n=166)** | **NO**  **(Survivors)**  **(n=564)** |  |
| DM (n,%) | 95 (57.2%) | 312 (55.3%) | 0.663 |
| HTN (n,%) | 85 (51.2%) | 264 (46.8%) | 0.319 |
| CKD (n,%) | 32 (19.3%) | 60 (10.6%) | 0.003 |
| CVA (n,%) | 8 (4.8%) | 27 (4.8%) | 0.986 |

Abbreviations: **CKD:** Chronic kidney disease, **CVA:** Cerebrovascular accident, **DM:** Diabetes mellitus, **HTN:** Hypertension.

**Supplementary table 3 : The value of NLR and PLR for survivors and non-survivors of COVID-19**

| **Parameters** | **Survivors**  **(n=564)** | **Non-survivors**  **(n=166)** | **p-value** |
| --- | --- | --- | --- |
| NLR (mean ± SD) | 5.70 (3.20-11.12) | 10.08 (5.35-18.78) | <0.001 |
| PLR (mean ± SD) | 207.48 (134.12-339.24) | 289.13 (143.57-485.59) | 0.001 |

Abbreviations: **NLR:** Neutrophil-lymphocyte ratio, **PLR:** Platelet-lymphocyte ratio.
